# Phase Transformation of VO_**2**_ Nanoparticles Assisted by Microwave Heating

**DOI:** 10.1155/2014/841418

**Published:** 2014-02-04

**Authors:** Phatcharee Phoempoon, Lek. Sikong

**Affiliations:** ^1^Department of Mining and Materials Engineering, Faculty of Engineering, Prince of Songkla University, Hat Yai 90112, Thailand; ^2^CENE Center of Excellence in Nanotechnology for Energy, Prince of Songkla University, Hat Yai 90112, Thailand

## Abstract

The microwave assisted synthesis nowadays attracts a great deal of attention. Monoclinic phase VO_2_ (M) was prepared from NH_4_VO_3_ and H_2_C_2_O_4_ · 2H_2_O by a rapid microwave assisted technique. The synthesis parameters, microwave irradiation time, microwave power, and calcinations temperature were systematically varied and their influences on the structure and morphology were evaluated. The microwave power level has been carried out in range 180–600 W. TEM analysis demonstrated nanosized samples. The structural and morphological properties were measured using XRD, TEM, and thermal analyses. The variations of vanadium phase led to thermochromic properties.

## 1. Introduction

Vanadium dioxide undergoes a transition temperature is around 68°C. It is a change of crystallographic structure, from monoclinic (M phase, semiconductor) at temperatures below *T*_c_ to tetragonal (R phase, metal) at temperatures above *T*_c_ [1]. As a result, VO_2_ materials have been considered for a variety of potential applications such as energy-efficient window coatings [2], thermal sensors [3], cathode materials for reversible lithium batteries [4], and electrical and infrared light switching device [5, 6]. There are also several methods that have been reported to synthesize VO_2_ nanocrystalline, including physical vapor deposition, aqueous reduction, ion implantation, chemical vapor deposition, sol-gel [7], excimer-laser-assisted metal organic deposition (ELAMOD) [8], magnetron sputtering [9], chemical vapor deposition (CVD) [10], pulsed laser deposition (PLD) [11], vacuum evaporation [12], and hydrothermal [13], and reduction hydrolysis [14]. However this technique needs long reaction time, high temperature treatment, and complicated processing.

Microwave assisted synthesis is a novel method of synthesis [15] and it is a rapidly developing research area. In this system, an understanding of the microwave interaction with materials has been based on the concept of dielectric heating and the resonance absorption due to rotational excitation [16]. The microwave method provides the advantage of uniform, rapid, and volumetric heating. In microwave synthesis, the growth rate of products is very high for small particle sizes and, nearly always, the product exhibits a narrow particle size distribution as a consequence of fast homogenous nucleation [17]. The benefits expected from the use of microwaves are not only energy saving due to volumetric heating but also the prospects of developing novel materials with improved properties that cannot be fabricated by conventional methods. A variety of materials like carbide, nitride, sulfides [15, 18], and complex oxides have been synthesized by microwave irradiation method. These materials are of industrial and technological importance [16].

In view of this, the pure phase VO_2_ (M) was fabricated via a simple solution-based process [19] and we employed microwave irradiation method for evaporation of solution. It is shown that the irradiation durations required are remarkably short. Various operational parameters have been varied systematically so that a deeper understanding of the mechanism of nanoparticle formation is obtained. These parameters include the influence of temperature, concentration of reagents, digestion time, thermal treatments using conventional oven, and the effect of microwave treatment. The objective of this study is to characterize the influence of microwave field on the sequence of phase transformations in VO_2_ (M). The properties of the synthesized VO_2_ (M) powders were investigated by X-ray diffraction (XRD), differential scanning calorimeter (DSC), differential thermal analysis (DTA), scanning electron microscope (SEM), and transmission electron microscopy (TEM).

## 2. Experimental

### 2.1. Synthesis of VO_2_ (M) Nanoparticles

The VO_2_ (M) nanoparticles were synthesized by simple solution-based process using NH_4_VO_3_ the source of vanadium and oxalic acid (H_2_C_2_O_4_ · 2H_2_O) as reducing agent. All of the chemical reagents used in the experiments were of analytical grade. A 0.5 g portion of NH_4_VO_3_ was firstly stirred with 50 mL of hot water (60°C). Then oxalic acid was added to the suspension during heating. The pH value was measured as 2. The solution turned yellow and then blue, indicating the reduction of V^5+^ to V^4+^. The mixture was stirred continuously for 30 min and then the above solution was dried by microwave oven. Microwave irradiation was varied between 180 and 600 W. The dry solid is amorphous at this stage. Therefore, in comparison with the microwave method, the sample was also prepared by the conventional oven heating method that was dried below 100°C. This material can be converted to VO_2_ (M) by calcination in an inert environment. The dried powder was calcined at different temperatures (400–700°C) with a heating rate of 5°C min^−1^ in a flow of nitrogen gas for 1 h.

### 2.2. Characterization

The morphology of nanostructures was investigated using a FEI Quanta 400 scanning electron microscope (SEM) and a JEOL JEM 2010 transmission electron microscope (TEM). X-ray diffraction (XRD) was carried out on a Phillip X'Pert MPD. Differential scanning calorimetry (DSC) experiment of VO_2_ powders was performed using Perkin-Elmer DSC-7 with a heating rate at 5°C min^−1^. DTA measurements were carried out on Perkin-Elmer DTA-7. The samples were heated from 100°C to 700°C at a heating rate of 5°C min^−1^ in nitrogen atmosphere.

## 3. Result and Discussion

### 3.1. Effect of Microwave Treatment on DTA

**Figure 1:**
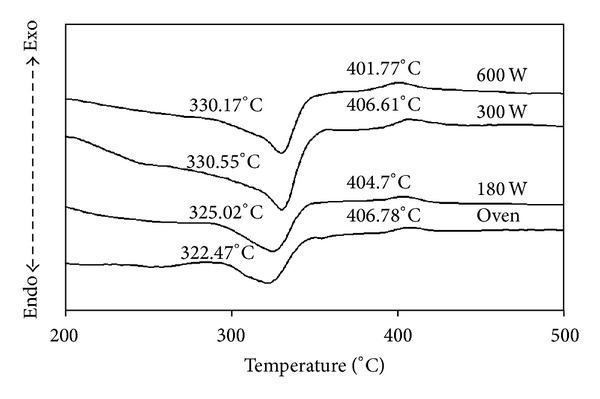
DTA curves of vanadium oxide powders prepared by drying method. A comparison between the oven heating at 100°C and the microwave heating performed at different irradiation powers of 180 W, 300 W, and 600 W.

DTA curves of the precursor are shown in Figure 1. The DTA data indicate that the weight loss of the precursor began at about 322°C and ended at about 407°C. There were four peaks of losing weight on the DTA curve, which demonstrated the formation of two intermediates in the thermolysis process of the compound. There were endothermic peaks at 322.47, 325.02, 330.55 and 330.1°C, respectively, on the DTA curve of the precursor which were due to thermolysis effects. The exothermic peaks synthesized by using oven and microwave powers of 180, 300, and 600 W occurred at 406.78, 404.70, 406.61, and 401.77°C, respectively. This temperature correlated with the crystallization of V_2_O_3_ and V_4_O_7_ powders. According to the XRD pattern in Figure 3, the crystallization of vanadium dioxide powders began around 400°C.

**Figure 2:**
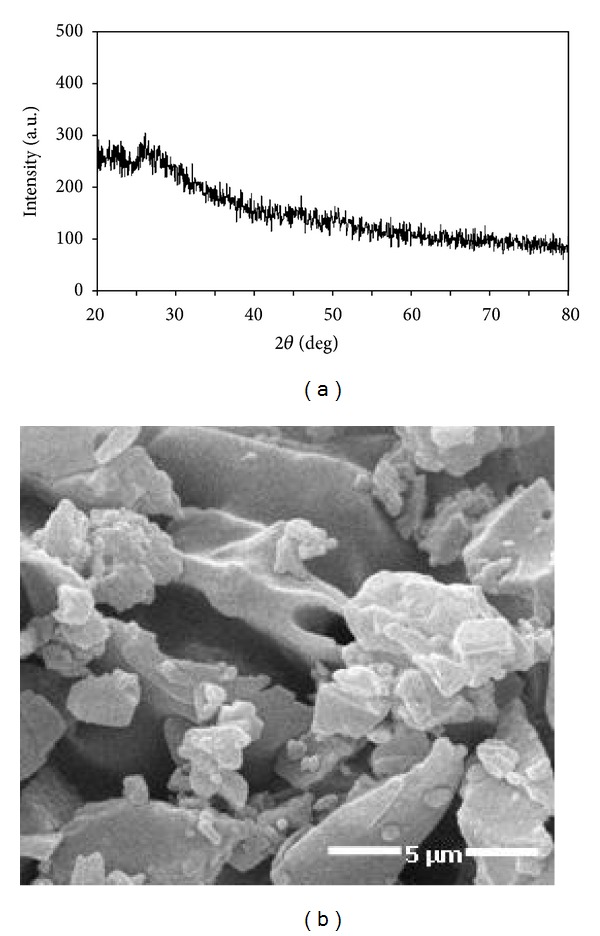
(a) XRD patterns and (b) SEM image of as-synthesized vanadium oxide sample prepared by the microwave heating at 300 W.

XRD patterns of vanadium dioxide powders dried by microwave irradiation powers at 300 W are shown in Figure 2(a). The as-prepared vanadium dioxide powder, even after drying at 300 W, was found to be vanadium oxalate (VOC_2_O_4_) in amorphous form. The possible reactions in the solution and the decomposition of the intermediate are listed as follows [20, 21],

(1) 2NH4VO3+4C2H2O4  ⟶(NH4)2[(VO)2(C2O4)3]+2CO2+4H2O

(2) (NH4)2[(VO)2(C2O4)3]  ⟶2VOC2O4+2NH3+CO+CO2+H2O

(3) $$\begin{array}{c} \text{VO}{\text{C}}_{2}{\text{O}}_{4}⟶\text{V}{\text{O}}_{2}+\text{CO}+\text{C}{\text{O}}_{2} \end{array}$$

### 3.2. Effect of Microwave Treatment, Oven Treatment, and Calcinations Temperature on VO_2_ (M) Phase

XRD patterns of vanadium dioxide powders dried at microwave irradiation powers and calcined at different temperature for 1 h are shown in Table 1. For all samples prepared at 400–500°C are V_2_O_3_, V_4_O_7_, and V_3_O_5_. The monoclinic phase of VO_2_ (M) was formed via drying with microwave at 300 W and calcined at temperatures of 500 and 600°C. Increasing microwave irradiation power to 600 W, VO_2_ (M) peak intensity decreased and the monoclinic VO_2_ (M) converted to corundum V_2_O_3_ and V_3_O_5_, while the V_2_O_3_ and V_3_O_5_ were formed via oven drying and calcination at 600°C. In addition, when the calcination temperature rose to 700°C, the mixed phase of VO_2_ (M) and V_4_O_7_ was found.

As compared to the conventional oven method, the microwave method is faster because in microwave method the waves coupled directly with the molecules that are heating, leading to a rapid increase in the temperature [16].

**Figure 3:**
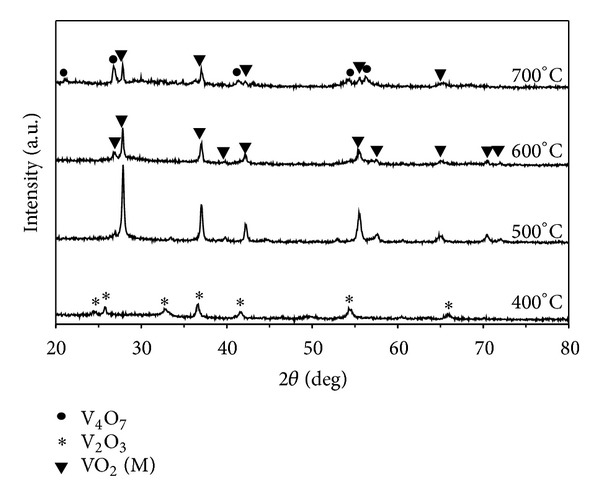
XRD patterns of vanadium oxide powders prepared by microwave heating at 300 W and then calcinations at different temperatures for 1 h.

Typical XRD patterns are shown in Figure 3. Based on the above temperature analysis, the calcination temperatures of 400, 500, 600, and 700°C were designed for the preparation of nanocrystalline VO_2_ (M). At the same microwave power (300 W), it could be seen that the peaks VO_2_ (M) were observed only at calcination temperature above 500°C, indicating that crystalline VO_2_ (M) particles were successfully prepared after calcinations. The mechanism of phase transformation is independent of the method of heating [22].

**Figure 4:**
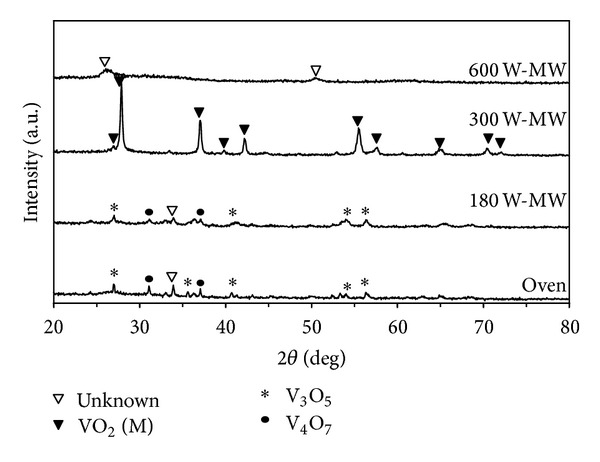
XRD patterns of vanadium oxide powders prepared by the microwave heating at different microwave irradiation powers and calcination at the temperature of 500°C for 1 h.

Figure 4 shows that the XRD patterns of vanadium oxide powders prepared by the microwave heating at different microwave irradiation powers and calcination at the temperature of 500°C for 1 h. It can be seen that the diffraction peaks of samples obtained after microwave irradiation at 300 W and calcination at temperature of 500°C can be indexed to monoclinic VO_2_ and the monoclinic structure can be able to convert into another crystalline material when treated with higher microwave power.

The XRD pattern can be readily indexed as the monoclinic crystalline phase of VO_2_ (M) with calculated lattice constant *a* = 5.76476 Å, *b* = 4.53173 Å, *c* = 5.38200 Å and *a* = 5.75853 Å, *b* = 4.50136 Å, *c* = 5.39756 Å, respectively, which corresponds to VO_2_ (M) (JCPDS 01-082) (crystallographic information is shown in Table 2). Only monoclinic phase was detected from XRD pattern via microwave heating at 300 W and calcination at 500 and 600°C. However, a mixture of vanadium oxide is obtained when using another condition.

### 3.3. Effect of Microwave Treatment on Morphology

**Figure 5:**
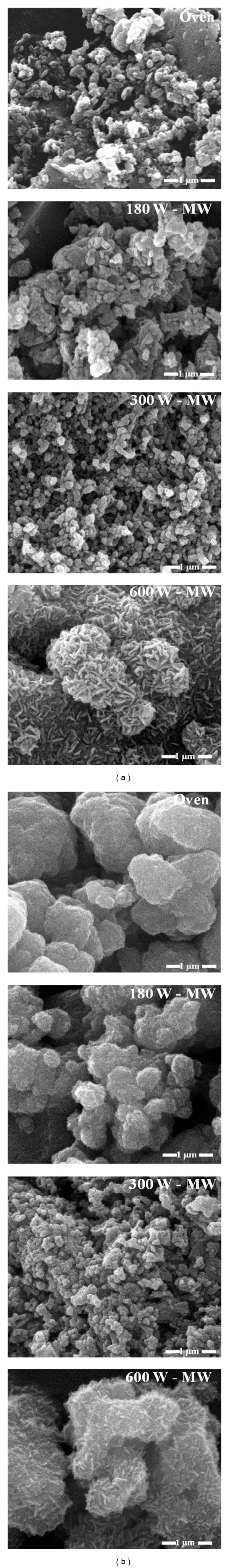
SEM micrographs of vanadium oxide powders prepared by the oven heating at 100°C and the microwave heating at different microwave irradiation powers 180–600 W for 15 min and then calcined at (a) 400°C and (b) 500°C for 1 h.

Figure 5 depicts SEM pictures of vanadium oxide powder dried by the oven heating and the microwave heating at different wattages (180–600 W) and calcination at temperatures of 400°C and 500°C. From the micrograph, it was observed that the particles were agglomerated but were almost uniform in size. The particle morphology observed was spherical with uneven shapes in powders dried by microwave irradiation at 300 W. It seems that the smallest particle size was obtained with the microwave heating at 300 W while the agglomeration of small flake-like particles formed at power 600 W. Furthermore, the influence of higher calcination temperature on grain growth was clearly observed. The microwave treatment reduces the particle size and increases the homogeneity of the materials. This is due to the enhanced surface enrichment due to thermal agitation of liquid molecules in microwave field. The apparent change in the material yields improvement which results in the evolvement of new material phases. Microwave treatment is thus a rapid approach that has the capability to control the particle shape and particle size [23]. It was concluded that microwave treatment improved the monoclinic crystalline structure of the vanadium dioxide.

**Figure 6:**
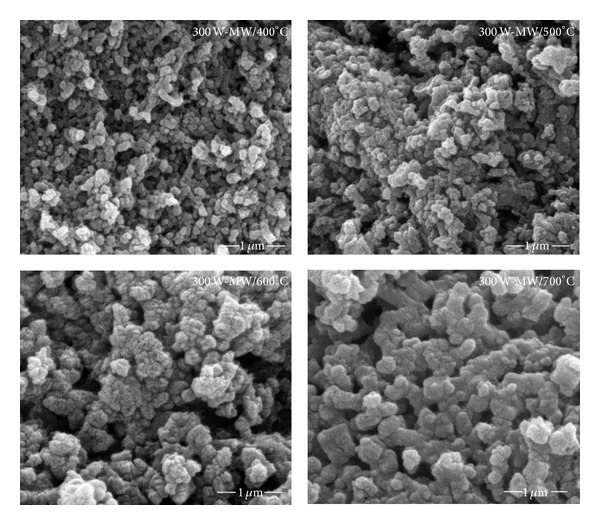
SEM micrographs of vanadium oxide powders prepared by the microwave heating at 300 W for 15 min and calcination at temperature of 400–700°C for 1 h.

Scanning electron micrographs of the vanadium oxide obtained by microwave heating at power 300 W also show a grain coarsening with the increasing calcination temperatures (Figure 6). The micrograph of powder calcined at 400°C has a particle with a 0.1-0.2 *μ*m size distribution. Between 400 and 500°C, there is no significant morphology change. The vanadium oxide prepared at calcination temperatures of 400 and 500°C exhibits similar morphological features (e.g., size and shape). On increasing the calcination temperature to 600°C, recrystallization and agglomeration of the primary particles occurs. It is noteworthy to mention that the crystallite size of the vanadium oxide does increase after 500°C.

**Figure 7:**
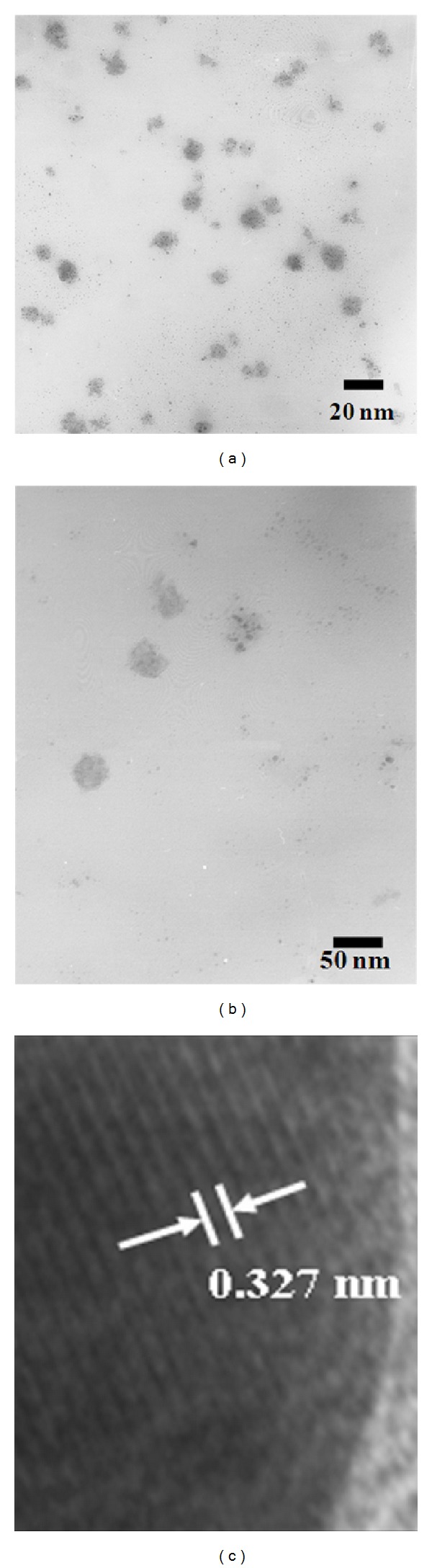
TEM micrographs of VO_2_ (M) powder prepared by microwave irradiation power 300 W and calcination at temperature 500°C for 1 h.

TEM micrographs of VO_2_ (M) nanopowders are shown in Figure 7. Figures 7(a) and 7(b) show that the powder consisted mainly of spherical particles and the particle size was found to be less than 50 nm.  Figure 7(c) shows the lattice-resolved TEM images. The fringe is around 0.327 nm, which corresponds to the *d* spacing of the (011) plane of monoclinic VO_2_ (M) phase [24, 25].

### 3.4. Effect of Oxalic Acid Loading on Phase Transformation

**Figure 8:**
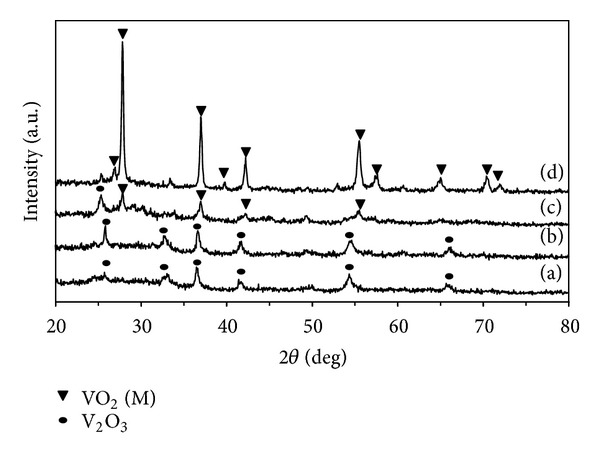
XRD patterns of vanadium oxide powders prepared by calcination of the 300 W microwave heated samples at 400°C for 1 h with different molar ratios of NH_4_VO_3_ and oxalic acid: (a) 2 : 2.7, (b) 2 : 2.8, (c) 2 : 2.9, and (d) 2 : 3.

**Figure 9:**
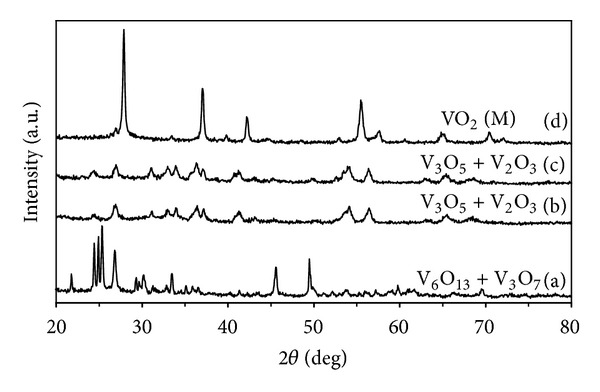
XRD patterns of vanadium oxide powders prepared by calcination of the 300 W microwave heated samples at 500°C for 1 h with different molar ratios of NH_4_VO_3_ and oxalic acid: (a) Nonoxalic acid, (b) 2 : 2.6, (c) 2 : 2.7, and (d) 2 : 2.8.

In Figure 8, it was found that VO_2_ (M) was formed by calcinations at temperature 400°C, where the molar ratio of NH_4_VO_3_ and oxalic acid was kept at 2 : 3. Oxalic acid could not totally reduce NH_4_VO_3_ to VO_2_ (M) when the ratio was less than 2 : 3. It should be noted that oxalic acid was necessary for the formation of VO_2_ (M). With the Microwave heating of the NH_4_VO_3_ solution without oxalic acid, sample powders formed exclusively without any VO_2_ (M) (Figure 9(a)). The suitable amount of oxalic acid plays the important role as coordinating ligand or reducing agent helping the formation of VO_2_ (M). It was found that the molar ratio of 2 : 2.8 is suitable for the sample prepared by microwave heating at power 300 W and then calcined at 500°C for 1 hour (Figure 9(d)).

**Figure 10:**
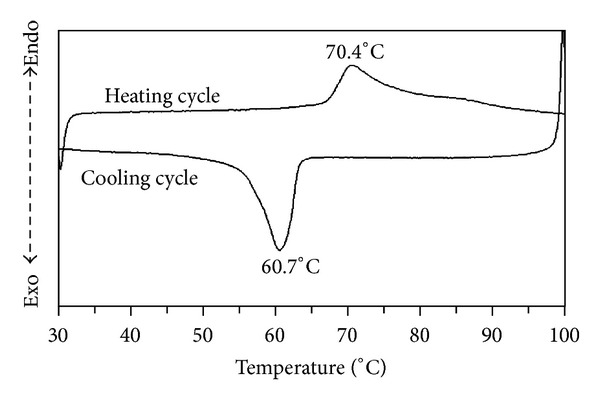
DSC curves of VO_2_ (M) powders prepared by calcination of the 300 W microwave heated samples at 500°C.

The phase transition temperature of VO_2_ (R/M) was determined by observing the change of some property during heating process and the hysteresis property was defined by the corresponding change that occurred during heating and cooling processes [26]. The DSC curves in Figure 10 described the phase transition of pure phase VO_2_ (M) and showed the hysteresis of the phase transition between phase VO_2_ (M) and VO_2_ (R) during heating and cooling. During heating process, the endothermic peak appeared at 70.40°C and the exothermic peak appeared at 60.70°C during cooling process. These transition temperatures shifted from the ordinary figures of 67-68°C due to the agglomeration of the synthesized nanopowders which required more thermodynamic energy to transform phase from monoclinic to rutile.

## 4. Conclusions

This is the first time that preparation of nanocrystalline VO_2_ (M) has been achieved by a microwave irradiation-assisted process using the precursor vanadium and oxalic acid as reducing agents. Thus, it would be concluded that the applied microwave powers also do affect the formation of monoclinic phase structure of VO_2_ synthesized powders. In microwave synthesis, the growth rate of products is very high for small particle sizes. It indicates that microwave has improved the crystallinity of the particles. This method can reduce the synthesis time. The adoption of the microwave method offers chances to generate new material structures that cannot be obtained from conventional methods. Microwave irradiation at power 300 W and calcination in an inert gas atmosphere at temperature of 500°C for 1 h promote the uniformly formation of VO_2_ (M). Morphology and structure of nanostructured VO_2_ (M) have been controlled by microwave irradiation power and dosage of an oxalic acid.

## Figures and Tables

**Table 1:** Summary of grain size and phases of vanadium oxides after calcinations of the microwave assisted samples at different temperatures.

Temperature of calcinations (°C)	Microwave power	Phase from XRD	Grain size calculated using Scherrer equation (nm)
400	Oven	V_2_O_3_ + V_4_O_7_	56.6
	180 W	V_2_O_3_	42.7
	300 W	V_2_O_3_	42.5
	600 W	V_2_O_3_	24.3

500	Oven	V_2_O_3_ + V_3_O_5_	42.2
	180 W	V_2_O_3_ + V_3_O_5_	41.5
	300 W	VO_2_ (M)	41.6
	600 W	—	—

600	Oven	V_2_O_3_ + V_3_O_5_	42.6
	180 W	—	—
	300 W	VO_2_ (M)	41.6
	600 W	V_2_O_3_ + V_3_O_5_	42.5

700	Oven	VO_2_ (M) + V_4_O_7_	41.6
	180 W	—	—
	300 W	VO_2_ (M) + V_4_O_7_	55.5
	600 W	VO_2_ (M) + V_4_O_7_	41.5

**Table 2:** Lattice parameters of VO_2 _(M) analyzed by XRD.

Lattice parameters/materials	*a* (Å)	*b* (Å)	*c* (Å)	*c*/*a*
VO_2_ (M) 300 W-MW 500°C	5.76476	4.53173	5.38200	0.9336
VO_2_ (M) 300 W-MW 600°C	5.75853	4.50136	5.39756	0.9373
VO_2_ (M) (JCPDS 01-082)	5.75290	4.52630	5.38250	0.9356
